# Analytically Confirmed Intoxications Involving MDMB-CHMICA from the STRIDA Project

**DOI:** 10.1007/s13181-016-0584-2

**Published:** 2016-09-16

**Authors:** Matilda Bäckberg, Luiza Tworek, Olof Beck, Anders Helander

**Affiliations:** 1 Swedish Poisons Information Centre, SE-171 76 Stockholm, Sweden; 2 Department of Laboratory Medicine, Karolinska Institutet, Stockholm, Sweden; 3 Karolinska University Laboratory, Clinical Pharmacology, Stockholm, Sweden

**Keywords:** Drug exposure, New psychoactive substances, Spice, Synthetic cannabinoid receptor agonists, Synthetic cannabinoids

## Abstract

**Introduction:**

About a decade ago, synthetic cannabinoids (SC) started to appear as recreational drugs on the new psychoactive substance (NPS) market. This report from the STRIDA project describes analytically confirmed intoxications involving MDMB-CHMICA (methyl-2-(1-(cyclohexylmethyl)-1*H*-indol-3-ylcarbonylamino)-3,3-dimethylbutanoate), a SC that was first detected in 2014.

**Study Design:**

This is an observational case series of patients from Sweden with suspected NPS exposure presenting in emergency departments and intensive care units. The results of retrospective serum and urine toxicological analysis were compared with clinical signs reported during consultation with the Poisons Information Centre and retrieved from medical records.

**Methods:**

Clinical and bioanalytical data in nine acute intoxications associated with MDMB-CHMICA during 2014–2015 are presented. The patients were aged 23–62 (median 34) years, and eight were men. MDMB-CHMICA (parent compound) was analytically confirmed in serum samples, using a liquid chromatography–high-resolution mass spectrometry multi-component method.

**Results:**

Of the nine MDMB-CHMICA-positive patients, eight had a Poisoning Severity Score (PSS) of 2 or 3, and five were monitored in the intensive care unit and all patients survived. Development of seizures and deep unconsciousness were common features. All cases except one also tested positive for other NPS and/or classical psychoactive compounds, hampering the possibility to establish a causal relationship between drug and toxic symptoms. MDMB-CHMICA was also identified in seven drug materials donated by the patients.

**Conclusions:**

The association with severe adverse reactions in nine acute analytically confirmed intoxication cases involving MDMB-CHMICA is consistent with other reports of serious toxicity linked to this substance, suggesting that MDMB-CHMICA might be a particularly harmful SC.

## Introduction

About a decade ago, synthetic cannabinoid receptor agonists (synthetic “cannabinoids,” SC) started to appear as recreational drugs in the form of laced herbal smoking blends. These substances, of which many were originally developed as drug candidates and pharmacological probes, are nowadays collectively referred to as “spice,” from the brand name of an early herbal smoking blend. The SC are typically made available through the rapidly expanding online-sale market for new psychoactive substances (NPS) [1–3]. Attempts to control the open sale of yet unclassified SC and other NPS through substance-specific or generic regulations have led to market adaptations and the appearance of hundreds of novel chemical substances since 2008 [4–6].

The SC are functionally similar to Δ^9^-tetrahydrocannabinol (THC), the principal psychoactive constituent in cannabis, targeting the main central nervous system receptor (CB_1_) in the endocannabinoid system [7]. Compared with THC, which is a partial CB_1_ receptor agonist, many novel SC show full receptor agonist effect and also a significantly greater affinity, making them much more potent and potentially more toxic [8, 9]. CB receptor overstimulation is therefore a likely cause for serious adverse events related to SC use [10, 11]. However, adverse side effects unrelated to the intended psychoactive targets are an inherent risk with untested NPS and may also be important in cases of SC toxicity [12].

**Fig. 1:**
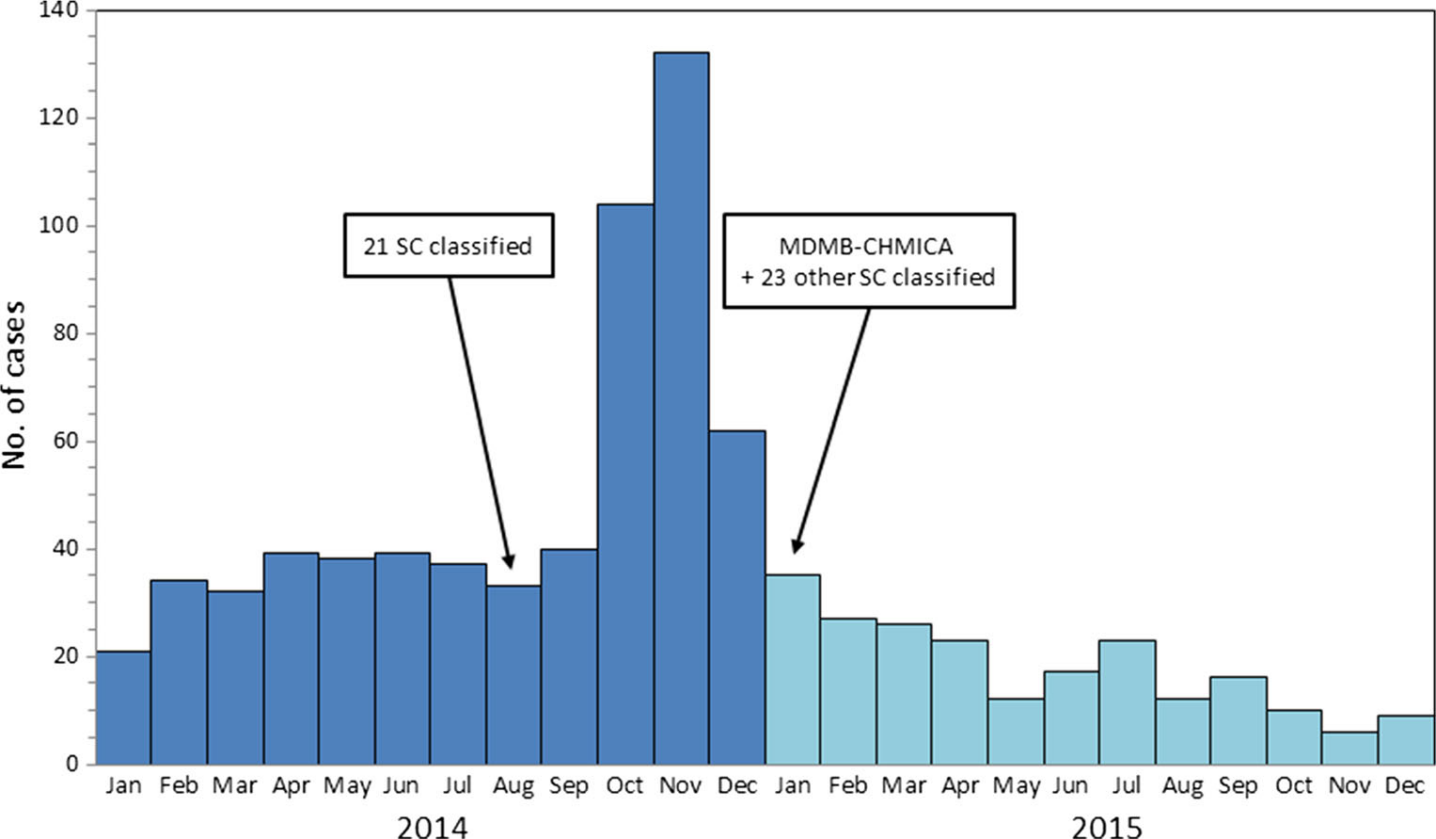
Monthly statistics from the Swedish Poisons Information Centre of cases (telephone calls mainly from hospital caregivers, but also the public) regarding suspected intoxications by synthetic cannabinoids (SC,“spice”). Times for Swedish classification of SC as being "hazardous to health" are also indicated

Since 2014, cases of severe SC toxicity have been reported with increasing frequency in many parts of the world. During the fall of 2014, SC were involved in at least 15 deaths and 600 patients requiring medical treatment in Russia [13]. During the same time, concerns about increases in SC-related harms were also raised in Sweden. In October and November 2014, the number of calls related to SC toxicity to the Swedish Poisons Information Centre equaled the total number of SC-related calls in 2013 (∼250 calls), and most days comprised 5 %, and occasionally even 10 %, of all calls from hospitals (Fig. 1). Similarly, in the USA, the number of poison center calls related to SC use more than tripled during the spring of 2015 with 15 deaths and more than 300 potentially life-threatening cases [14].

**Fig. 2:**
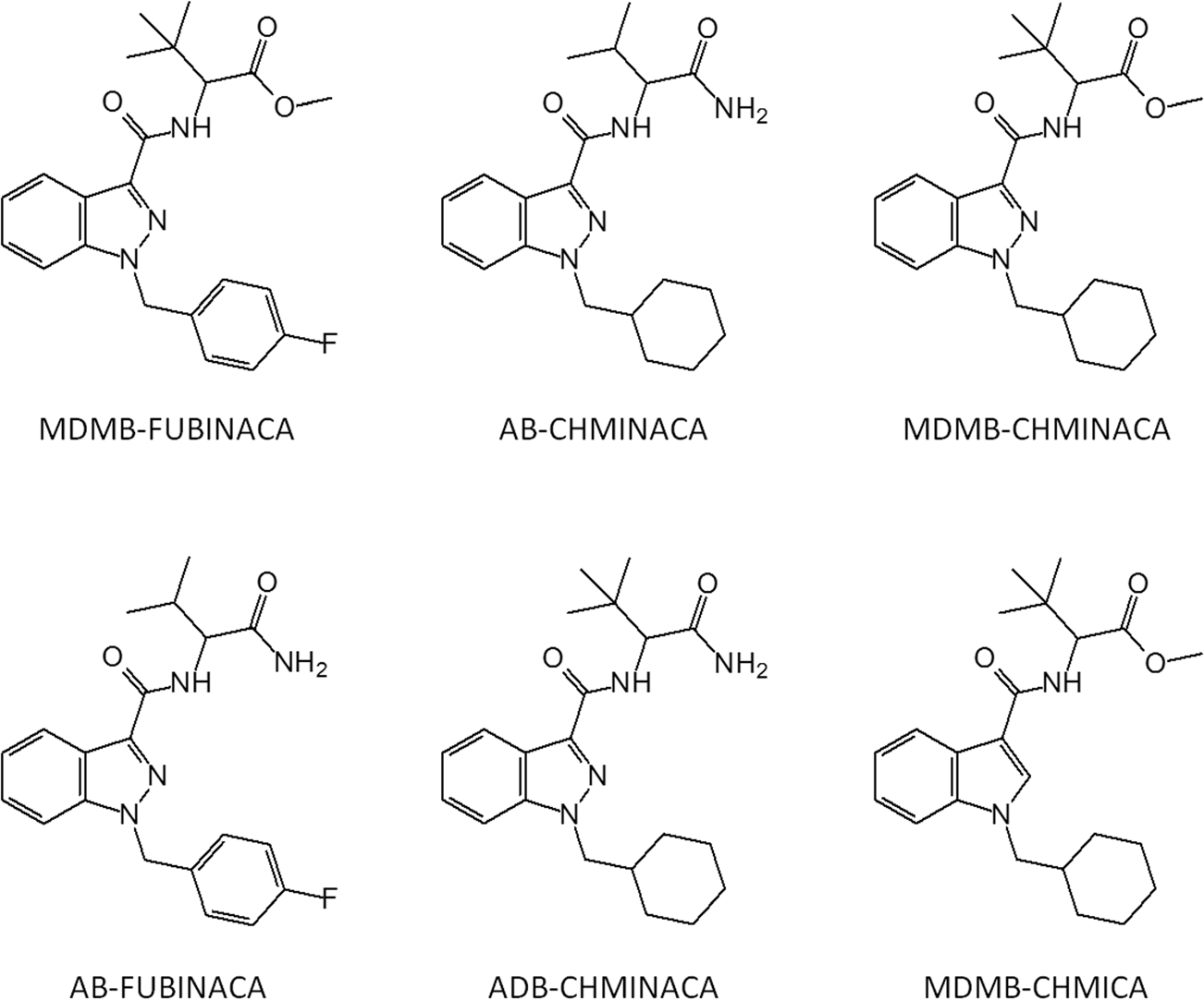
Chemical structures of synthetic cannabinoids (“spice”) recently associated with severe toxicity across the world. MDMB-FUBINACA (Russia), AB-FUBINACA (USA), AB-CHMINACA (USA), ADB-CHMINACA (USA), MDMB-CHMINACA (Pfizer patent “indazol” compound), MDMB-CHMICA (Europe; present study)

The seemingly increasing risk of harm related to SC use is likely due to a greater toxicity of the novel variants [14], many of which showing more complex molecular structures compared with THC and the “first generation” SC (e.g., JWH-018) (Fig. 2). The Russian cases of severe SC toxicity in 2014 were suspected to involve MDMB-FUBINACA (also known as MDMB N-Bz F), whereas ADB-CHMINACA, AB-CHMINACA, and AB-FUBINACA have been associated with the US cases [13, 15–19]. MDMB-CHMICA is another structurally similar SC (Fig. 2) that has attracted growing attention, since it was first reported to the European Monitoring Centre for Drugs and Drug Addiction (EMCDDA) and Europol in the fall of 2014, due to the connection with cases of severe toxicity and fatalities [20–25].

This case series from the STRIDA project presents clinical and bioanalytical data in nine non-fatal acute intoxications analytically confirmed to involve MDMB-CHMICA (methyl-2-(1-(cyclohexylmethyl)-1*H*-indol-3-ylcarbonylamino)-3,3-dimethylbutanoate), one of several SC involved in a Swedish “spice epidemic” in the fall of 2014.

## Methods

### Patient Inclusion Criteria

The STRIDA (an acronym in Swedish) project is an ongoing collaboration since 2010 between the Swedish Poisons Information Centre and the Karolinska University Laboratory (Stockholm), aiming to monitor the occurrence and health hazards of NPS in Sweden. When clinicians contact the Poisons Information Centre (a nationwide 24/7 service to hospital caregivers and the public) for treatment advice in acute intoxication cases with suspected exposure to NPS presenting in emergency departments (ED) and intensive care units (ICU), they are also encouraged to sample blood and urine for free analysis of psychoactive drug contents [26, 27]. The samples are analyzed for a large number of NPS and classical drugs-of-abuse (currently >300 substances are covered). Data on clinical features are collected by the Poisons Information Centre during consultations and reviewed retrospectively from medical records. In each case, the severity of intoxication was graded retrospectively using the Poisoning Severity Score (PSS) [28]. The level of consciousness was defined by the Reaction Level Scale (RLS) or the Glasgow Coma Scale (GCS) [29, 30].

At the time of the Swedish “spice epidemic” in the fall of 2014, reference material for analytical confirmation of MDMB-CHMICA was initially missing. Thus, the present cases of confirmed MDMB-CHMICA exposure were selected from the pool of samples from STRIDA patients (882 patients from October 2014 to October 2015) and analyzed retrospectively, because there was a reason to suspect MDMB-CHMICA poisoning (e.g., the substance had been mentioned in connection with the intoxication or the patient brought packets labeled with and/or analytically confirmed to consist of MDMB-CHMICA).

The STRIDA project is conducted in accordance with the Helsinki Declaration and is approved by the regional ethical review board (No. 2013/116–31/2).

### Laboratory Analysis

Laboratory investigations of urine specimens for contents of NPS and classical psychoactive substances were done according to published procedures, using both immunochemical screening assays and multicomponent methods based on liquid chromatography combined with tandem (LC–MS/MS) or high-resolution (LC–HRMS) mass spectrometry [27, 31, 32]. The analysis of SC including MDMB-CHMICA was performed in serum (parent compounds only) using LC–HRMS, after a solvent protein precipitation procedure. Specimens were stored at −20 °C until taken for analysis.

Reference materials for SC, including the novel substances shown in Fig. 2 (except MDMB-FUBINACA, for which an early warning system formal notification on its first detection in Europe was issued in January 2016), were obtained from Chiron AS (Trondheim, Norway) and Cayman Chemical Co (Ann Arbor, MI, USA). The limit of detection (LOD) for MDMB-CHMICA was 0.6 ng/mL and the lower limit of quantification (LLOQ) was 1.25 ng/mL.

Drug materials (e.g., powder, tablets, and herbal smoking mixtures) brought to hospital by the patients were sometimes sent to the laboratory together with the serum and urine samples. These items were forwarded to the Swedish Medical Product Agency (Uppsala) for analytical investigation, using LC–quadrupole-time-of-flight–MS/MS (LC–QTOF–MS/MS) and nuclear magnetic resonance (NMR) spectroscopy [33].

## Results

### Clinical Data and Laboratory Investigations

Data on patient demographics, clinical features, and bioanalytical results in the nine selected intoxication cases involving MDMB-CHMICA are presented in Tables 1 and 2. The patients were aged 23–62 (median 34, mean 38.6) years, and all but one were males. With the exception of cases #1 and #2 (Table 1), of which case #1 is described in more detail below, all cases were seemingly unrelated and originated from different parts of Sweden.

**Table 1:** Laboratory and clinical data in nine acute intoxication cases analytically confirmed to involve MDMB-CHMICA exposure

Case no	Sex/age (years)	Date	Reported or suspected substance(s)	ROA (drug form)	Sampling time (hours after admission)	MDMB-CHMICA conc. in serum (ng/mL)^b^	Other substances detected in urine and/or blood (likely treatment related)	Main clinical characteristics	PSS
1	M/34	2015/Apr	Krokodil, spice, bath salt	nas/inh	1	3.8	(Desmethyldiazepam)	GCS 4, seizure episodes, vomiting, dilated pupils, SaO_2_ 88 %, BPM 150, BP 90/55 mmHg, pH 7.26, standard-bicarbonate 21 mmol/L, P-lactate 3.5 mmol/L, P-creatinine 100 μmol/L	2
2	M/41	2015/Apr	Krokodil, spice, bath salt	nas/inh	2.5	3.4	Diphenidine, HDMP-28, (buprenorphine, desmethyldiazepam, temazepam)	GCS 4, seizure episodes, miotic pupils, RR 10/min, SaO_2_ 86 %, BPM 150, BP 112/57 mmHg, pH 7.24, pCO_2_ 9.0 kPa (67.5 mmHg), standard-bicarbonate 24 mmol/L, P-creatinine 102 μmol/L, P-myoglobin 431 μg/L	3
3	M/44	2014/Oct	NM-2201 (Banana burst incense blend)	inh	3	35.9	Desmethyldiazepam, EtG/EtS, oxazepam, temazepam	RLS 1, delirium, dilated pupils, P-creatinine 112 μmol/L	1
4	M/28	2014/Nov	MMB-CHMINACA, NM-2201, THJ-018 (BlueBerry blitz incense blend), 5-HTP	–	0.5	Detected <LLOQ	Pregabalin	GCS 12, extreme agitation on admission, delirium, dilated pupils, RR 8/min, BPM 150, BP 141/81 mmHg, pH 7.3, pCO_2_ 7.0 kPa (52.5 mmHg)	3
5	M/59	2014/Dec	THJ-018, 5-HTP, ethanol	po and inh	Promptly	21.5	5F-APINACA	RLS 4, agitation, delirium, cyanosis, RR 25/min, SaO_2_ 70.5 %, BP 64 mmHg, temperature 33.3 °C (91.9 °F), pH (v) 7.27, pCO_2_ 4.1 kPa (30.8 mmHg), standard-bicarbonate 15.1 mmol/L, B-lactate 4.1 mmol/L, tHb 172 g/L, B-Na 159 mmol/L, B-creatinine 236 μmol/L, B-CK 82.2 μkat/L (4932 U/L)	2
6^a^	M/23	2015/Aug	MMB-CHMINACA, Flubromazepam	– (Powder)	1	3.4	Flubromazepam, ethanol^^^, (diazepam)	GCS 3, seizure episodes, vomiting, dilated pupils, RR 29/min (temporary apnea), SaO_2_ 83 %, BPM 167, pH 7.14, pCO_2_ 7.6 kPa (57 mmHg), pO_2_ 24.0 kPa (180 mmHg), standard-bicarbonate 19 mmol/L, B-lactate 9.5 mmol/L, P-creatinine 110 μmol/L	3
7	F/24	2015/Sep	MMB-CHMINACA, alprazolam, buprenorphine	Repeated inh	Promptly	86.4	Buprenorphine, THC-COOH	RLS 5, agitation, seizure episodes, BPM 110, BP 140/70 mmHg, pH 7.27, pCO_2_ 5.9 kPa (44.2 mmHg), pO_2_ 11.7 kPa (87.8 mmHg), P-lactate 6.9 mmol/L	2
8	M/32	2015/Sep	MMB-CHMINACA	– (powder)	0.5	26.2	AMB-FUBINACA, clonazolam, 4-OH-alprazolam, buprenorphine, THC-COOH	GCS 14, dilated pupils, BPM 30	2
9	M/62	2015/Oct	MMB-CHMINACA, THJ-018, Flubromazolam	– (powder)	Promptly	15.6	THJ-018	RLS 5, seizure episodes, vomiting, P-creatinine 143 μmol/L	2

Data show MDMB-CHMICA-positive STRIDA cases collected from October 2014 to October 2015

*ROA* route of administration, *PSS* Poisoning Severity Score [28], *po* peroral, *nas* nasal (snorted), *inh* inhalation (smoked), − information missing, *5-HTP* 5-hydroxytryptophan, *EtG/EtS* ethyl glucuronide/ethyl sulfate (ethanol metabolites), *HDMP-28* methylnaphthidate, *6-MAM* 6-monoacetylmorphine, *^* data retrieved from medical records, *GCS* Glasgow Coma Scale, *RLS* the Reaction Level Scale [29, 30], *SaO*_*2*_ oxygen saturation, *BPM* beats per minute, *BP* blood pressure, *P* plasma, *RR* respiratory rate, *B* blood, *tHb* total hemoglobin, *CK* creatinine kinase

^a^Only serum available

^b^LOD = 0.6 ng/mL and LLOQ 1.25 ng/mL

**Table 2:** Clinical features documented on admission to hospital and/or collected during telephone consultation with the Swedish Poisons Information Centre in nine analytically confirmed intoxications involving MDMB-CHMICA

Clinical features	*n* = 9
Elevated P-creatinine (≥100 μmol/L)	6
Dilated pupils	5
Seizures	5
Tachycardia (≥100/min)	5
Deep unconsciousness^a^ (RLS ≥5; GCS ≤4)	5
Respiratory depression (RR ≤10/min, SaO_2_ ≤ 90 %)	5
Elevated B/P-Lactate (≥2.2 mmol/L)	4
Agitation	3
Delirium	3
Vomiting	3

^a^May be influenced by co-exposure of sedatives, postictal state, etc

*RLS* the Reaction Level Scale [30], *GCS* Glasgow Coma Scale, *RR* respiratory rate, *SaO*_*2*_ oxygen saturation, *B* blood, *P* plasma

In eight of the nine cases, both serum and urine specimens sampled at the same time within 2.5 h after arrival to hospital were available for analysis. MDMB-CHMICA (parent compound) could not be identified in any urine sample. However, the serum samples from eight patients contained MDMB-CHMICA in the range 3.4–86.4 (median 18.6, mean 24.5) ng/mL, whereas the concentration was below the LLOQ in one case (Table 1). The patient showing by far the highest substance concentration (86 ng/mL), being twofold higher than the second highest level, reported previous experience of smoking “spice,” but had smoked for the first times a new SC named “MMB-CHMINACA” (an incorrect name for MDMB-CHMICA often used by internet vendors and in drug discussion forums) every ∼4 h starting ∼18 h before arrival to hospital.

All nine cases also tested positive for other NPS and/or classical drugs of abuse (Table 1), although some of these substances may have been treatment related. Accordingly, case #1 was considered to be an MDMB-CHMICA mono-intoxication, because diazepam which had been given during acute treatment was the only other substance detected. In addition to MDMB-CHMICA, four other SC (5F-APINACA, AMB-FUBINACA, NM-2201, and THJ-018) were identified in the serum samples (Table 1).

Besides the serum and urine samples, four patients had brought to hospital a total of seven drug materials that were also forwarded to the laboratory and later confirmed by LC–QTOF–MS/MS and NMR to contain MDMB-CHMICA (Table 3). Two of the materials were unlabeled, two were labeled “MMB-CHMINACA” (i.e., MDMB-CHMICA), whereas three had labels that referred to other SC.

**Table 3:** Results from analysis of NPS products brought to hospital by patients testing positive for MDMB-CHMICA in serum

Case no	Product labeling	Sample form	Analysis technique	Major SC detected	Minor SC content	Trace content^a^	Tentative SC^a^
3	NM-2201 (Banana burst incense blend)	Dried herbs	LC–QTOF–MS/MS and NMR	MDMB-CHMICA			
4	MMB-CHMINACA	White powder	LC–QTOF–MS/MS and NMR	MDMB-CHMICA			
4	NM-2201, THJ-018 (BlueBerry blitz incense blend)	Dried herbs	LC–QTOF–MS/MS and NMR	MDMB-CHMICA	THJ-018		5F-PB-22
5	THJ-018	Residues only	LC–QTOF–MS/MS	THJ-018, MDMB- CHMICA (molar ratio unknown)^a^			BB-22
8	MMB-CHMINACA	White powder	LC–QTOF–MS/MS and NMR	MDMB-CHMICA		AMB-FUBINACA	1-(cyclohexylmethyl)-1*H*-indole-3-carbonyl)valine (or a structural isomer)
8	Unlabeled	White powder	LC–QTOF–MS/MS and NMR	MDMB-CHMICA		AMB-FUBINACA	1-(cyclohexylmethyl)-1*H*-indole-3-carbonyl)valine (or a structural isomer)
8	Unlabeled	White powder	LC–QTOF–MS/MS and NMR	MDMB-CHMICA			1-(cyclohexylmethyl)-1*H*-indole-3-carbonyl)valine (or a structural isomer)

^a^Content not verified by NMR

*LC–QTOF–MS/MS* liquid chromatography-quadrupole-time-of-flight–tandem mass spectrometry, *NMR* nuclear magnetic resonance spectrometry, *SC* synthetic cannabinoids

In six drug items, MDMB-CHMICA constituted the only or major psychoactive compound; four of these were white powders and two were herbal smoking mixtures (Table 3). Although quantitative 1H-qNMR was not performed, the NMR data suggested high purity of MDMB-CHMICA in all powders (≥95 %) and no excipients were detected (personal communication, K-H Jönsson, Swedish Medical Product Agency). In addition to MDMB-CHIMCA, traces of other SC (not verified by NMR) were found in five products (THJ-018, 5F-PB-22, AMB-FUBINACA, BB-22, and 1-(cyclohexylmethyl)-1*H*-indole-3-carbonyl)valine). There was one additional dried herbal product that contained MDMB-CHMICA belonging to a patient that tested negative to MDMB-CHMICA and therefore excluded from the present study; however, in this smoking blend, 5F-APINACA was present in much higher amount than MDMB-CHMICA.

The most relevant clinical signs and symptoms in the nine patients testing positive for MDMB-CHMICA are listed in Table 2. Most features, including development of seizures and deep unconsciousness (Reaction Level Scale [RLS] ≥5; Glasgow Coma Scale [GCS] ≤4), were indicated to be more frequent in the patients showing the lowest serum MDMB-CHMICA concentrations (<10 ng/mL).

All patients were transported to hospital by ambulance, and police assistance was needed in two cases. Five patients were treated with diazepam on ambulance arrival, during transport, and/or in hospital, and three patients needed sedation with propofol of which two were intubated. Four patients were monitored in the ED or in a medical observation unit for <8 h, while the other five required ICU monitoring of which one (case #5) needed intensive care for 4 days and was not discharged home until day 6. The resuming patients were all discharged within 24 h from admission.

### Case Series of MDMB-CHMICA Exposure

Three male in-patients (cases #1, #2, and one test-negative case that was excluded from the present study) at the same drug addiction treatment center had ingested an unknown substance claimed to be “krokodil”, “spice”, or “bath salt”. The route of drug administration was never clarified. Within a few minutes after intake, all three started vomiting, lost consciousness, and had tonic-clonic seizures, according to reports from the treatment center staff. The paramedics in the first arriving ambulance administered 5 mg diazepam rectally to each patient, and they were then transported to two different hospitals. Both ED teams contacted the Poisons Information Centre for consultation.

Patient #1 was a 34-year-old man with a history of poly-substance abuse. During transport to hospital, he was still seizing despite given diazepam, was bleeding from bites to his tongue, and was deeply unconscious (GCS 4). He received an additional 30 mg of diazepam intravenously. His airways were clear and he breathed spontaneously, receiving 15 L/min oxygen via a face mask. He was febrile (38 °C), tachycardic (150/min), and the systolic BP was 90–85 mmHg.

Upon arrival to the ED, his initial laboratory parameters in plasma (pH 7.26, pCO_2_ 7.1 kPa [53 mmHg], pO_2_ 8.0 kPa [60 mmHg], base excess −4.4 mmol/L, standard bicarbonate 21.0 mmol/L, lactate 3.5 mmol/L, potassium 3.3 mmol/L, and creatinine 100 μmol/L) showed a mild acidosis. Within 1 h of observation, he woke up and was communicable although not completely lucid. His vital signs, including the body temperature, had normalized. The patient opposed continued care at hospital and was discharged back to the treatment center after an uneventful 8-h observation period.

In the biological samples that were collected 1 h after ED admission, MDMB-CHMICA and desmethyldiazepam were detected; the latter is a metabolite of diazepam and its presence was likely treatment related. In this and the second related case, serum MDMB-CHMICA concentrations of 3.8 and 3.4 ng/mL, respectively, were found, whereas the substance was not detectable in the third related case (hemolytic serum sample). The sample from the second case also contained other psychoactive substances (Table 1).

## Discussion

The nine selected poisonings involving MDMB-CHMICA presented in this case series indicate that the novel SC can cause serious acute harm, thereby being in sharp contrast to what is typical for cannabis (THC). In this respect, the case presented in greater detail (case #1) was especially decisive, because no other substances were detected, and life-threatening symptoms developed within minutes of drug intake. It should be pointed out that MDMB-CHMICA is only one of several potent SC associated with an outbreak of SC toxicity (called a “spice epidemic” in the media) in Sweden during the fall of 2014. It is also interesting to note that six of the nine intoxications involving MDMB-CHMICA occurred, after the substance had been regulated in Sweden in January 2015 as being “hazardous to health” [34].

According to the information from the EMCDDA and Europol, the first European early warning report on MDMB-CHMICA originated from Hungary in August 2014. A discussion thread devoted to the substance on a Swedish internet forum, albeit then incorrectly named “MMB-CHMINACA,” was started in mid-September 2014. The first seizure of MDMB-CHMICA by the Swedish Police occurred in October the same year, as did the first analytically confirmed poisoning in the STRIDA project. During the fall of 2014, the substance was detected in nine autopsy cases in Sweden and in at least two of those considered as the cause of death [23, 35]. During 2014–2015, MDMB-CHMICA has also been linked to deaths in Norway [25] and to deaths and cases of serious toxicity in Germany, Poland, and the UK [20, 21, 24, 36].

The adverse effects noted in the present case series are consistent with findings from other cases of MDMB-CHMICA intoxication [21, 24, 36, 37] and with those reported in poisonings involving other potent novel SC [15–19, 38]. Seizures, deep unconsciousness, and agitated delirium were common clinical findings, all being potentially life-threatening. A common laboratory finding was the occurrence of a raised plasma creatinine concentration (≥100 μmol/L). However, the elevations occurred early in the clinical course and were relatively mild, suggesting dehydration, muscular exertion, and possibly rhabdomyolysis, rather than nephrotoxicity, as the cause.

Besides their high potency (i.e., receptor affinity), the severe toxicity associated with novel SC may in part be related to the new practice of selling products as pure powders, which increases the risk of overdose, instead of as ready-for-use herbal smoking mixtures. The MDMB-CHMICA powder samples analyzed in this study were demonstrated to be of high purity, being consistent with discussions on drug forums that they are intended for making “stock solutions” [39].

A somewhat remarkable observation was that the patients showing a relatively low MDMB-CHMICA concentration in blood were indicated to display a more severe toxidrome, compared to the patients with the highest substance levels. Also in previous studies, however, extremely low concentrations have been reported in fatal cases and severely ill patients [20, 21, 23, 25, 40]. Contrary, on admission to hospital, one of the patients (case #7) in the present case series had a relatively high MDMB-CHMICA concentration in serum (86 ng/mL), which is about eightfold higher than in previous publications on related fatalities. This patient admitted recurrent smoking over the previous ∼18 h, which might have resulted in drug accumulation and could explain the high level reached. The patient had developed seizure-like symptoms a few minutes after the last smoking session, and the blood sample was estimated to have been collected about 1 h after the onset of symptoms. A similarly high serum MDMB-CHMICA concentration, in a subject not displaying any toxicity symptoms, has been reported once before [21]. This may result from tolerance building after chronic use of SC [10, 21, 41]. Posts at discussions online also indicate that frequent use of MDMB-CHMICA quickly leads to tolerance, since some users stated smoking 2 g/day within a few weeks, and even withdrawal symptoms are described [42].

Similar to THC, the SC are lipophilic compounds that are extensively metabolized, but knowledge about metabolic patterns is often limited, and so is the availability of reference materials. A common analytical strategy to confirm SC use is therefore measurement of the parent compounds in blood specimens, rather than of urinary metabolites which are the standard procedure for THC (i.e., testing for THC-COOH). Nevertheless, due to a generally shorter detection window in blood after drug intake, blood samples need to be collected closer to exposure [43, 44], and, due to the high potency of novel SC and hence low substance concentrations, may require highly sensitive analytical methods [20, 21, 25, 40]. In some SC studies involving investigation of metabolites, their presence has mainly been used as additional (confirmatory) support of drug exposure [16, 19, 37, 45].

The present cases represent only a small part of the Poisons Information Centre calls on SC presented in Fig. 1. The substances involved in the “spice epidemic” in Sweden at the end of 2014 are still under investigation, and since then, other potent and potentially toxic SC have been introduced on the recreational drugs market. However, it should also be pointed out that after the peak of harmful cases in the end of 2014, the number of contacts to the Poisons Information Centre related to SC has shown a steady decline.

As demonstrated in Fig. 2, MDMB-CHMICA and the other SC are structurally similar to the substances described in a 2009 Pfizer patent comprising over 700 indazole derivatives showing CB_1_ activity [46]. MDMB-CHMICA is the indole variant of the patented substance MDMB-CHMINACA which has a CB_1_ receptor affinity ∼400 times that of THC [13]. The high toxicity associated with MDMB-CHMICA use illustrates that the indazole core structure of the Pfizer patent can be substituted for an indole, without apparent loss of effect and it is probable that it could also be substituted for yet other building blocks, e.g., benzimidazole. Thus, the Pfizer patent could form the basis for a staggering number of SC for the NPS market [4].

Limitations of this study include missing or unreliable information about the amount of drug taken, the route of administration, and the time passing between intake and arrival in hospital and sampling of blood, as well as the small number of cases. Also, unknown compound stability and inter-individual variability in drug metabolism are confounding factors for MDMB-CHMICA identification. In addition, as in previous case series on NPS from the STRIDA project, poly-drug use was found to be common, implying possible risks for interferences by other psychoactive substances and making it difficult to link substance concentration levels to severity grade as well as confirming what clinical signs are specifically related to MDMB-CHMICA. Finally, because the way clinical information is collected is not standardized between hospitals, information on less obvious signs and symptoms may have been missed.

## Conclusions

The association with severe adverse reactions in nine acute analytically confirmed poisonings cases involving MDMB-CHMICA in Sweden in 2014–2015 is consistent with other reports of serious toxicity linked to this substance, supporting that MDMB-CHMICA is one of the more toxic SC found so far on the NPS market.

## References

[1] EMCDDA. EU drug markets report 2016—in-depth analysis. 2016. Available at: http://www.emcdda.europa.eu/system/files/publications/2373/TD0216072ENN.PDF.

[2] Fattore L, Fratta W (2011). Beyond THC: the new generation of cannabinoid designer drugs. Front Behav Neurosci.

[3] Seely KA, Lapoint J, Moran JH, Fattore L (2012). Spice drugs are more than harmless herbal blends: a review of the pharmacology and toxicology of synthetic cannabinoids. Prog Neuropsychopharmacol Biol Psychiatry.

[4] Advisory Council on the Misuse of Drugs. ‘Third generation’ synthetic cannabinoids. 2014. Available at: https://www.gov.uk/government/uploads/system/uploads/attachment_data/file/380161/CannabinoidsReport.pdf.

[5] Shanks KG, Dahn T, Behonick G, Terrell A (2012). Analysis of first and second generation legal highs for synthetic cannabinoids and synthetic stimulants by ultra-performance liquid chromatography and time of flight mass spectrometry. J Anal Toxicol.

[6] Kikura-Hanajiri R, Kawamura NU, Goda Y (2014). Changes in the prevalence of new psychoactive substances before and after the introduction of the generic scheduling of synthetic cannabinoids in Japan. Drug Test Anal.

[7] Wiley JL, Marusich JA, Huffman JW (2014). Moving around the molecule: relationship between chemical structure and in vivo activity of synthetic cannabinoids. Life Sci.

[8] Fantegrossi WE, Moran JH, Radominska-Pandya A, Prather PL (2014). Distinct pharmacology and metabolism of K2 synthetic cannabinoids compared to Delta(9)-THC: mechanism underlying greater toxicity?. Life Sci.

[9] Gurney SM, Scott KS, Kacinko SL, Presley BC, Logan BK (2014). Pharmacology, toxicology, and adverse effects of synthetic cannabinoid drugs. Forensic Sci Rev.

[10] Castaneto MS, Gorelick DA, Desrosiers NA, Hartman RL, Pirard S, Huestis MA (2014). Synthetic cannabinoids: epidemiology, pharmacodynamics, and clinical implications. Drug Alcohol Depend.

[11] Tait RJ, Caldicott D, Mountain D, Hill SL, Lenton S (2016). A systematic review of adverse events arising from the use of synthetic cannabinoids and their associated treatment. Clin Toxicol (Phila).

[12] Centers for Disease Control and Prevention (CDC) (2013). Acute kidney injury associated with synthetic cannabinoid use—multiple states, 2012. MMWR Morb Mortal Wkly Rep.

[13] Shevyrin V, Melkozerov V, Nevero A, Eltsov O, Shafran Y, Morzherin Y (2015). Identification and analytical characteristics of synthetic cannabinoids with an indazole-3-carboxamide structure bearing a N-1-methoxycarbonylalkyl group. Anal Bioanal Chem.

[14] Law R, Schier J, Martin C, Chang A, Wolkin A (2015). Notes from the field: increase in reported adverse health effects related to synthetic Cannabinoid use—United States, January–May 2015. MMWR Morb Mortal Wkly Rep.

[15] Centers for Disease Control and Prevention (CDC) (2013). Notes from the field: severe illness associated with synthetic cannabinoid use—Brunswick, Georgia, 2013. MMWR Morb Mortal Wkly Rep.

[16] Schwartz MD, Trecki J, Edison LA, Steck AR, Arnold JK, Gerona RR (2015). A common source outbreak of severe delirium associated with exposure to the novel synthetic cannabinoid ADB-PINACA. J Emerg Med.

[17] Shanks KG, Clark W, Behonick G (2016). Death associated with the use of the synthetic cannabinoid ADB-FUBINACA. J Anal Toxicol.

[18] Trecki J, Gerona RR, Schwartz MD (2015). Synthetic cannabinoid-related illnesses and deaths. N Engl J Med.

[19] Tyndall JA, Gerona R, De Portu G, Trecki J, Elie MC, Lucas J (2015). An outbreak of acute delirium from exposure to the synthetic cannabinoid AB-CHMINACA. Clin Toxicol (Phila).

[20] Adamowicz P (2016). Fatal intoxication with synthetic cannabinoid MDMB-CHMICA. Forensic Sci Int.

[21] Angerer V, Franz F, Schwarze B, Moosmann B, Auwarter V (2016). Reply to ‘sudden cardiac death following use of the synthetic cannabinoid MDMB-CHMICA’. J Anal Toxicol.

[22] EMCDDA. European drug report 2016: trends and developments. 2016. Available at: http://www.emcdda.europa.eu/system/files/publications/2637/TDAT16001ENN.pdf.

[23] Kronstrand R, Tyrkko E, Lindstedt M, Roman M, editors. MMB-CHMINACA blood concentrations in recreational users and fatal intoxications. Poster presentation at 53rd The International Association of Forensic Toxicologists meeting 2015; 2015 August 30th to September 4th; Firenze.

[24] Seywright A, Torrance HJ, Wylie FM, McKeown DA, Lowe DJ, Stevenson R. Analysis and clinical findings of cases positive for the novel synthetic cannabinoid receptor agonist MDMB-CHMICA. Clin Toxicol (Phila) 2016:1–6. 10.1080/15563650.2016.1186805 27213960

[25] Westin AA, Frost J, Brede WR, Gundersen PO, Einvik S, Aarset H (2016). Sudden cardiac death following use of the synthetic cannabinoid MDMB-CHMICA. J Anal Toxicol.

[26] Helander A, Beck O, Hägerkvist R, Hultén P (2013). Identification of novel psychoactive drug use in Sweden based on laboratory analysis—initial experiences from the STRIDA project. Scand J Clin Lab Invest.

[27] Helander A, Bäckberg M, Hultén P, Al-Saffar Y, Beck O (2014). Detection of new psychoactive substance use among emergency room patients: results from the Swedish STRIDA project. Forensic Sci Int.

[28] Persson HE, Sjöberg GK, Haines JA, Pronczuk de Garbino J (1998). Poisoning severity score. Grading of acute poisoning. J Toxicol Clin Toxicol.

[29] Starmark JE, Stålhammar D, Holmgren E (1988). The reaction level scale (RLS85). Manual and guidelines. Acta Neurochir (Wien).

[30] Starmark JE, Stålhammar D, Holmgren E, Rosander B (1988). A comparison of the Glasgow coma scale and the reaction level scale (RLS85). J Neurosurg.

[31] Al-Saffar Y, Stephanson NN, Beck O (2013). Multicomponent LC-MS/MS screening method for detection of new psychoactive drugs, legal highs, in urine-experience from the Swedish population. J Chromatogr B Analyt Technol Biomed Life Sci.

[32] Bäckberg M, Lindeman E, Beck O, Helander A (2015). Characteristics of analytically confirmed 3-MMC-related intoxications from the Swedish STRIDA project. Clin Toxicol (Phila).

[33] Johansson M, Fransson D, Rundlöf T, Huynh NH, Arvidsson T (2014). A general analytical platform and strategy in search for illegal drugs. J Pharm Biomed Anal.

[34] Förordning om ändring i förordningen (1999:58) om förbud mot vissa hälsofarliga varor [Regulation of changing the regulation of certain goods hazardous to health]. 2014. Available at: http://www.notisum.se/rnp/sls/sfs/20141481.pdf.

[35] EMCDDA. EMCDDA–Europol Joint Report on a new psychoactive substance: methyl 2-[[1-(cyclohexylmethyl)indole-3-carbonyl]amino]-3,3-dimethylbutanoate (MDMB-CHMICA). 2016. Available at: http://www.emcdda.europa.eu/system/files/publications/2873/2016.4528_WEB.pdf.

[36] Hill SL, Najafi J, Dunn M, Acheampong P, Kamour A, Grundlingh J, et al. Clinical toxicity following analytically confirmed use of the synthetic cannabinoid receptor agonist MDMB-CHMICA. A report from the Identification Of Novel psychoActive substances (IONA) study. Clin Toxicol (Phila) 2016;54:638–43. 10.1080/15563650.2016.1190980 27251903

[37] Hermanns-Clausen M, Müller D, Kithinji J, Angerer V, Franz F, Eyer F, et al., editors. Abstract 17; Acute side effects after consumption of the novel synthetic cannabinoids AB-CHMINACA and MDMB-CHMICA. 36th International Congress of the European Association of Poisons Centres and Clinical Toxicologists (EAPCCT), Madrid, Spain, 2016. Clinical Toxicology, 54:4, 344–519, DOI: 103109/1556365020161165952; 2016.

[38] Adamowicz P, Gieron J. Acute intoxication of four individuals following use of the synthetic cannabinoid MAB-CHMINACA. Clin Toxicol (Phila) 2016;54:650–4. 10.1080/15563650.2016.1190016 27227269

[39] Flashback. Available at: https://www.flashback.org. Accessed May 2016.

[40] Abouchedid R, Thurtle N, Yamamoto T, J. H, Bailey G, Hudson S, et al., editors. Abstract 238; Analytical confirmation of the synthetic cannabinoid receptor agonists (SCRAs) present in a cohort of presentations with acute recreational drug toxicity to an Emergency Department (ED) in London, UK. 36th International Congress of the European Association of Poisons Centres and Clinical Toxicologists (EAPCCT), Madrid, Spain, 2016. Clinical Toxicology, 54:4, 344–519, DOI: 103109/1556365020161165952.

[41] Hermanns-Clausen M, Kneisel S, Szabo B, Auwarter V (2013). Acute toxicity due to the confirmed consumption of synthetic cannabinoids: clinical and laboratory findings. Addiction.

[42] Drugs-Forum–MDMB-CHMICA. Available at: https://drugs-forum.com/forum/showthread.php?t=253270. Accessed May 2016.

[43] Namera A, Kawamura M, Nakamoto A, Saito T, Nagao M (2015). Comprehensive review of the detection methods for synthetic cannabinoids and cathinones. Forensic Toxicol.

[44] Vikingsson S, Green H (2016). Putting designer drugs back in Pandora’s box: analytical challenges and metabolite identification. Clin Chem.

[45] Hermanns-Clausen M, Kithinji J, Spehl M, Angerer V, Franz F, Eyer F, et al. Adverse effects after the use of JWH-210—a case series from the EU Spice II plus project. Drug Test Anal 2016. 10.1002/dta.1936 26768345

[46] Pfizer patent WO2009106980—Indazole derivatives. 2009. Available at: https://patentscope.wipo.int/search/en/detail.jsf?docId=WO2009106980&recNum=1&maxRec=&office=&prevFilter=&sortOption=&queryString=&tab=PCTDescription. Accessed May 2016.

